# Serotonin Differentially Regulates L5 Pyramidal Cell Classes of the Medial Prefrontal Cortex in Rats and Mice

**DOI:** 10.1523/ENEURO.0305-17.2018

**Published:** 2018-02-06

**Authors:** Mary C. Elliott, Peter M. Tanaka, Ryan W. Schwark, Rodrigo Andrade

**Affiliations:** Department of Pharmacology, Wayne State University School of Medicine, Detroit, MI

**Keywords:** Corticofugal, development, intratelencephalic, prefrontal cortex, pyramidal cell, serotonin

## Abstract

The prefrontal cortex receives a dense serotonergic innervation that plays an important role in its regulation. However, how serotonin regulates different pyramidal and interneuron cell classes in this area is incompletely understood. Previous work in rats has shown that serotonin differentially regulates two classes of pyramidal cells in layer 5. It excites one class by activating 5-HT_2A_ receptors, whereas it more subtly modulates the integrative properties of the other by co-activating 5-HT_1A_ and 5-HT_2A_ receptors. Here we have used electrophysiological recordings, combined with retrograde labeling and morphological reconstruction, to show that the first cell class corresponds to long range corticofugal neurons and the second corresponds to intratelencephalic neurons. These results suggest that, in rats, serotonin facilitates subcortical output while more subtly modulating cortico-cortical and cortico-striatal output. Interestingly, these results obtained in rats differ from those previously reported for mouse prefrontal cortex. Therefore we reinvestigated the effects of serotonin in mice and confirmed that serotonin predominantly activates inhibitory 5-HT_1A_ receptors on long-range corticofugal cells. Thus serotonin exerts opposite effects on these cells in rats and mice. Finally, we determined whether cortical serotonin responsiveness in mice is regulated during development. Serotonin elicited predominantly depolarizing inward current responses during the early postnatal period, whereas inhibitory 5-HT_1A_ receptor-mediated responses did not become evident until the end of the second postnatal week. These results reveal commonalities as well as unexpected differences in the serotonergic regulation of long-range corticofugal and intratelencephalic neurons of layer 5 in rat and mouse.

## Significance Statement

Serotonin is thought to be an important regulator of prefrontal cortex function. Previous work has focused on layer 5 and identified heterogeneous effects of serotonin on pyramidal cells. This is consistent with the coexistence of long-range corticofugal and intratelencephalic pyramidal cells in this layer. However, the specific effects of serotonin reported in rats and mice differ, making it difficult to understand how serotonin regulates this area. Here we elucidate the effects of serotonin on both cell classes in rats and show that they differ from those in mice. These results extend our understanding of the effects of serotonin in layer 5 and suggest caution when extrapolating across rodents as we try to understand the functional role of serotonin in prefrontal cortex.

## Introduction

The cerebral cortex, including the prefrontal area, receives a dense serotonergic innervation originating predominantly from the dorsal raphe nucleus with a secondary contribution from the median raphe nucleus (Vertes, 1991; Van Bockstaele et al., 1993; Bang et al., 2012). Previous electrophysiological studies *in vivo* and *in vitro* have shown that the effects of serotonin on pyramidal cells and interneurons of cortex are highly variable, and this is thought to reflect the expression of varying serotonin receptor subtype combinations in different neuronal classes (Andrade and Beck, 2010; Andrade, 2011). However, exactly how serotonin regulates specific pyramidal cell and interneuron cell classes in cortex remains incompletely understood. Of particular interest is layer 5 (L5), which harbors two distinct subpopulations of pyramidal cells, one giving rise to long-range corticofugal projection and the other giving rise to intratelencephalic projections (Koester and O’Leary, 1993, reviewed by Molnar and Cheung, 2006; Molyneaux et al., 2007; Leone et al., 2008). These two populations are thought to differ not only in terms of their projections, but also in terms of their genomic regulation, electrophysiological properties, morphology, and neuromodulation (e.g. Molnar and Cheung, 2006; Hattox and Nelson, 2007; Dembrow et al., 2010; Avesar and Gulledge, 2012; Gee et al., 2012; van Aerde et al., 2015; Tasic et al., 2016).

Previous work in the rat medial prefrontal cortex (mPFC) has identified two distinct populations of pyramidal cells in L5 that show strikingly different modulation by serotonin (Beique et al., 2007). One of these cell populations expresses 5-HT_1A_ and 5-HT_2A_ receptors and responds to applications of serotonin with biphasic changes in excitability and a remodeling of its input-output relationship (Araneda and Andrade, 1991). The second, smaller, population expresses solely 5-HT_2A_ receptors and is strongly depolarized and excited by administration of serotonin. The relationship of these electrophysiologically and pharmacologically defined cell types to the long range corticofugal/intratelencephalic typology has not been addressed. More recent work in mouse mPFC has also reported a differential effect of serotonin on pyramidal cells of L5 (Avesar and Gulledge, 2012; Stephens et al., 2014). These studies showed that inhibitory 5-HT_1A_ receptors are expressed in both identified commissural (i.e., intratelencephalic) and corticopontine (i.e., long-range corticofugal) pyramidal cells of L5, whereas excitatory 5-HT_2A_ receptors are expressed predominantly on commissural pyramidal neurons. As a result, 5-HT selectively excites commissural/intratelencephalic L5 neurons. At the present time, it is difficult to mesh these results in rats and mice into a coherent understanding of the effects of serotonin in L5 of the mPFC. Therefore, in the current work, we have readdressed the effect of serotonin on pyramidal cells in L5 in rats and mice.

## Materials and Methods

Coronal slices from the mPFC were prepared from male and female Sprague-Dawley rats aged postnatal day 21 (P21) to P31 and male and female Swiss-Webster mice aged P7 to adult. Rats and mice were deeply anesthetized by inhalation using isoflurane and killed by decapitation. The brain was quickly removed from the skull, cooled in ice-cold Ringer (composition in mm: 119 NaCl, 2.5 KCl, 1.3 MgSO_4_, 2.5 CaCl_2_, 1 NaH_2_PO_4_, 26.2 NaHCO_3_, and 11 glucose) supplemented with 10 mm Hepes, and bubbled to saturation with 95% O_2_-5% CO_2_. In some experiments, brains were cooled and sectioned in a modified Ringer solution in which sodium was substituted with NMDG (composition in mm: 119 NMDG, brought to pH 7.3 with HCl, 2.5 KCl, 7 MgSO_4_, 0.5 CaCl_2_, 1 NaH_2_PO_4_, 26.2 NaHCO_3_, 22 glucose; 10 Hepes). The anterior portion of the brain was isolated, mounted to a stage with cyanoacrylate glue, then sliced (300-µm nominal thickness) using a Vibratome series 1000. Slices were transferred to a holding chamber that had an initial temperature of 35°C but was allowed to equilibrate to room temperature after the addition of slices. Slices spent a minimum of 1 h in the holding chamber before recording.

### Electrophysiological recordings

Whole-cell patch-clamp recordings were obtained from pyramidal neurons of the anterior cingulate or prelimbic regions of the mPFC. Cortical slices were transferred to a recording chamber on the stage of an upright microscope (Olympus BX50WI or Nikon E600), where they were continually perfused with Ringer at 31 ± 1°C bubbled to saturation with 95% O_2_-5% CO_2_. Slices were imaged using differential interference contrast (DIC), and pyramidal cells of L5 were identified using their position within the slice.

All recordings were performed using EPC 10 amplifiers (HEKA Instruments), and data were collected under the control of PatchMaster (HEKA Instruments). The recording pipettes were pulled from borosilicate glass (1.2 mm, Sutter Instruments) and filled with intracellular solution of the following composition (in mm): 120 KMeSO_4_, 5 KCl, 5 NaCl, 0.02 EGTA, 10 Hepes, 1 MgCl_2_, 10 phosphocreatine, 4 ATP Mg salt, 0.3 GTP Na salt, 10 inositol, pH 7.3. Electrode resistance ranged from 2.5 to 4 MΩ. For voltage-clamp experiments, cells were held at –60 mV. Cells referred to as type I pyramidal neurons seem to be less numerous than their type II counterparts in the mPFC. Therefore, to ensure an adequate sample size, cells of this type were not sampled at random, but instead were specifically targeted based on laminar position and soma size.

Calcium-activated afterhyperpolarization currents were triggered using a 100-ms depolarizing step to 10 mV to allow calcium into the cell as previously described (Villalobos and Andrade, 2010). The amplitude of the medium afterhyperpolarization current (I_mAHP_) and the slow afterhyperpolarization current (I_sAHP_) were measured at 10 and 400 ms after the completion of the depolarizing pulse, respectively. The hyperpolarization-activated cation current (I_h_) was triggered using a 2-s-long hyperpolarizing pulse from –60 mV to voltages ranging up to –110 mV. The amplitude and kinetics of I_h_ were estimated using the TraceFit component of the FitMaster analysis program (HEKA Instruments) during steps to –90 mV. In most cases, two exponential equations were needed to adequately fit I_h_ traces, and in the current work, we report values corresponding to the faster time constant. Spike frequency adaptation (SFA) was examined using 600-ms-long constant current depolarizing steps. For these experiments, we defined the spike frequency adaptation index (AI) as the ratio between the number of spikes in the final 300 ms of the 600-ms pulse to the number of spikes in the first 300 ms of the pulse in response to a depolarizing current injection between 500 and 700 pA. Previous studies have quantified spike frequency adaptation by comparing the third and last interspike interval (Hattox and Nelson, 2007; Chen et al., 2008). In the L5 pyramidal neurons of the mPFC, it was the cessation of spiking rather than the interspike interval that was the most notable feature. This feature is not well captured by comparing the third and last interspike interval; hence the current approach to quantifying accommodation. Input resistance was measured using a –200-pA current injection lasting 100 ms. The maximum voltage deflection was used to estimate the cell’s approximate input resistance.

All drugs were applied in the bath at the concentration stated in the text. To assess the effects of specific drugs on membrane current, cells were held at –60 mV, and the membrane current was recorded every 6 s, or every 30 s when also sampling I_sAHP_. Drugs were administered only once to each slice unless stated otherwise. When drugs were applied twice to the same slice, at least 10 min elapsed between applications. Mean current responses to serotonin represent the peak serotonin current with respect to the predrug baseline. All values are presented as mean ± SEM. Most chemicals were purchased from Sigma-Aldrich or Thermo Fisher Scientific. Tetrodotoxin (TTX) was obtained from Calbiochem-EMD, and apamin and WAY100135, from Tocris. MDL100907 was a gift of Dr. Kenner Rice (NIH/NIDDK).

### Cell reconstruction

Electrodes were filled with intracellular solution supplemented with 0.2%–1% biocytin. In this solution, KMeSO_4_ was reduced to 119.5 mm to maintain osmotic balance. After the experiment was completed, the electrode was left in place for a minimum of 20 min to allow for adequate filling. Slices were then fixed in 4% paraformaldehyde and refrigerated overnight, washed in PBS, and incubated overnight in streptavidin Alexa Fluor 568 conjugate (1:1000, Invitrogen) at 4°C with agitation. Slices were then washed three times in PBS and mounted. Filled neurons were imaged with an Olympus FluoView laser scanning confocal microscope equipped with a krypton laser and a 20× objective. To capture the full extent of the dendritic tree, most neurons were imaged in two partly overlapping stacks of equal depth. These stacks were stitched together using the “stack combiner” plug-in (MacBiophotonics) within ImageJ (NIH). More than 40 rat cells were filled and imaged, but only 11 of these yielded adequate morphologic reconstruction and the complete set of electrophysiological measurements necessary for analysis. Confocal image stacks from these 11 neurons were used to reconstruct the cells in 3D using Neurolucida (MBF Biosciences). The 3D reconstruction was then analyzed using Neuroexplorer (MBF Biosciences).

### Retrograde labeling

Male Sprague-Dawley rats (P19–P28) were injected with fluorescent latex beads (Retrobeads, LumaFluor) using a 1-µl syringe (Hamilton, 7001 KH). Four different locations were injected: mediodorsal thalamus (MD; 0.6 μl, stereotaxic coordinates, lateral, vertical, anterior/posterior from Bregma in mm, –2.8, –5.0, 0.8), dorsal raphe nucleus (0.5 μl, –8.0, –6.2, 0.1), contralateral cortex (CC; 0.2 μl, 2.0, –2.1, 0.5), and striatum (Str; 0.4 μl, –1.6, 4.6, 4.8). We recorded from striatal projecting cells in both hemispheres (ipsi- and contralateral to the injection site). This approach can be expected to greatly favor recording from intratelencephalic pyramidal cells. Injections were delivered over a 5- to 10-min window using manual control or a microsyringe injector and controller (World Precision Instruments, UMP2 Microsyringe Injector and Micro4 Controller). After the completion of the injection, the syringe needle was left in place for a minimum of 5 min before being withdrawn slowly. A minimum of 3 d elapsed between injection and the preparation of acute slices.

### Statistical analysis

Most statistical comparisons between groups used *t* tests. To generate an unbiased classification of the recorded neurons, a sample of 100 cells was sorted into groups using an unsupervised Bayesian classifier (Autoclass), as implemented in Autoclass@IJM (Achcar et al., 2009). Java Treeview was used to view the data and plot the results (Saldanha, 2004).

## Results

### Electrophysiological properties of pyramidal cells of L5 of the rat mPFC

**Figure 1.**
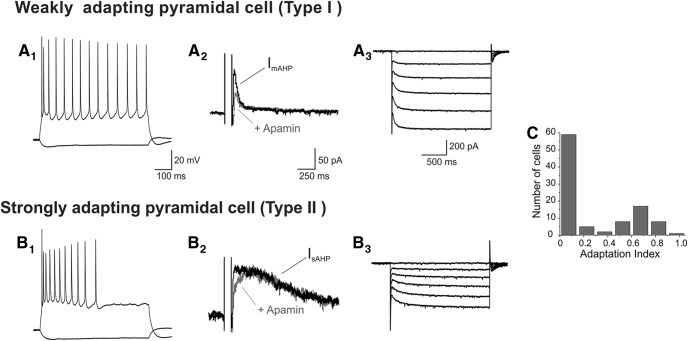
Electrophysiological properties define two pyramidal cell types in L5 of the rat mPFC. One of these corresponds to regular spiking neurons exhibiting limited firing frequency adaptation in response to constant depolarizing current injection (***A_1_***). These cells exhibit a prominent I_mAHP_ that is partially inhibited by apamin (100 nM, ***A_2_***) and a relatively fast activating I_h_ (***A_3_***) under voltage clamp. We have termed these cells type I, weakly adapting pyramidal cells. The second cell type exhibits strong spike frequency adaptation in response to constant depolarizing current injection (***B_1_***), a prominent apamin-insensitive I_sAHP_ (***B_2_***), and slow-activating I_h_ (***B_3_***). We have termed this second cell type II, strongly adapting pyramidal cells. ***A_2_***, ***A_3_***, ***B_2_***, ***B_3_***, Vh = –60 mV. Consistent with this typology, a histogram of spike frequency accommodation characteristics (as defined by the accommodation index) suggests a bimodal distribution (***C***).

We recorded from pyramidal neurons of L5 of the anterior cingulate and prelimbic subdivisions of the mPFC in brain slices derived from rats in the fourth and fifth postnatal weeks. As illustrated in Fig. 1, constant current depolarizing steps elicited repetitive spiking exhibiting variable spike frequency adaptation. Previous studies have shown that differences in spike frequency adaptation can be used to distinguish functionally distinct subpopulations of L5 pyramidal cells (Cho et al., 2004; Hattox and Nelson, 2007; Chen et al., 2008; Dembrow et al., 2010; van Aerde and Feldmeyer, 2015). Based on this criterion, most pyramidal cells in this area could be classified into two broad classes, regular spiking neurons with weak adaptation and regular spiking with strong adaptation (Fig. 1*A1* and 1*B1*). We quantified spike frequency adaptation by comparing the firing in the first and second halves of the depolarizing pulse (adaptation index, AI; see Methods). As illustrated in Fig. 1*C*, a histogram plotting the AI for 100 L5 pyramidal neurons recorded roughly sequentially produced a distribution consistent with the presence of two partly overlapping subpopulations.

Spike frequency adaptation in pyramidal cells is controlled by calcium-activated potassium currents and especially the slow calcium-activated afterhyperpolarization current (I_sAHP_, Madison and Nicoll, 1984; Schwindt et al., 1988, 1992). Consistent with this view, strongly adapting pyramidal cells expressed a robust I_sAHP_ when recorded in voltage clamp (Fig. 1*B2* ), whereas weakly adapting neurons generally exhibited little to no I_sAHP_ (Fig. 1*A2* ). Pyramidal cells express a second, faster calcium-activated potassium current that is carried by apamin-sensitive K_Ca_2.x–SK_Ca_ channels (Stocker et al., 1999; Villalobos et al., 2004) and contributes to the apamin-sensitive medium AHP current (I_mAHP_). In contrast to the marked difference in the amplitude of I_sAHP_ in strongly adapting and weakly adapting pyramidal cells, administration of apamin identified expression of this second calcium-activated potassium current in both cell types (Fig. 1*A2* and 1*B2*).

During our recordings, we noticed two additional differences that also appeared to distinguish strongly adapting and weakly adapting pyramidal cells in rat mPFC. First, weakly adapting cells tended to have a lower estimated input resistance than strongly adapting cells. Second, the hyperpolarization-activated nonselective cation current I_h_ elicited by stepping down from –60 mV also appeared to differ in these two cell types. Specifically, weakly adapting cells exhibited I_h_ currents exhibiting fast activation kinetics (Fig. 1*A3* ), suggesting the involvement of HCN1 subunits (Biel et al., 2009), whereas strongly adapting cells exhibited I_h_ currents with considerably slower activation kinetics (Fig. 1*B3* ).

### Sorting of L5 pyramidal cells using a Bayesian classifier

**Figure 2.**
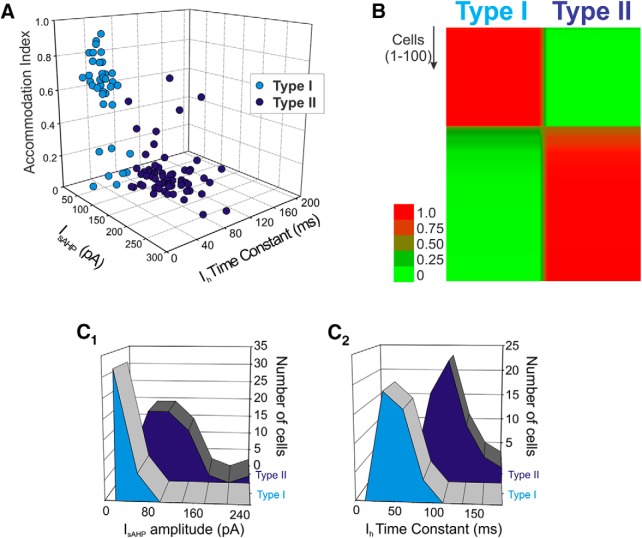
Sorting of L5 pyramidal cells. ***A***, Plotting the amplitude of I_sAHP_, the time constant of I_h_ and the AI cluster the cells in our sample into what appear to be two largely nonoverlapping subsets. ***B***, Autoclass, an unsupervised Bayesian classifier, also sorted the cells in our sample into two classes with the vast majority of cells sorting unambiguously to one of the two classes. The heat map depicts the probability that each cell tested in the current sample belongs to the type I or the type II classes. Probability is color-coded in a green-red scale as indicated in the side bar. After sorting, type I and type II cells, considered as groups, can be seen to differ in terms of the amplitude distribution for I_sAHP_ (***C_1_***) and the distribution of time constant for I_h_ (***C_2_***). ***C_1_***-***C_2_***: *n* = 100 cells.

The qualitative observations above suggested that we could use electrophysiological properties to distinguish two distinct subpopulations of neurons in L5 of the rat mPFC, a view that is consistent with previous findings by others (Dembrow et al., 2010; van Aerde and Feldmeyer, 2015). Therefore we next sought to explore this issue quantitatively. To that effect, we first plotted the AI, the amplitude of I_sAHP_, and the time constant for I_h_ for a group of 100 L5 pyramidal cells. As shown in Fig. 2*A*, this plot revealed what appeared to be two distinct clusters of neurons exhibiting little if any overlap in the 3D space defined by these three variables. These results support the idea of that these variables sort two discrete population of pyramidal cells. To quantitatively test this idea, we sorted our sample of L5 pyramidal cells using an unsupervised Bayesian classifier (Autoclass, Achcar et al., 2009) using the amplitude of I_sAHP_ and the I_h_ activation time constant as sorting variables. This unsupervised classifier determines the most probable number of classes (groups) in the data and assigns to each object (cell) the probability of belonging to a given class. Application of this classifier sorted the cells in our sample into two classes that we call type I (weakly adapting) and type II (strongly adapting). The results of this analysis are illustrated in the form of a heat map in Fig. 2*B*. In this graph each cell is represented by a horizontal line colored to denote, for each cell, the probability of it belonging to the type I and the type II classes, with red corresponding to a high probability and green a low probability. Autoclass unambiguously sorted the vast majority of cells into one of the two classes with very few ambiguous cases (Fig. 2*B*). To better understand the contributions of I_sAHP_ and I_h_ to the sorting of these two subpopulations of L5 pyramidal cells, we plotted the amplitude of I_sAHP_ and the time constant of activation for I_h_ for the two cells classes in our sample. As illustrated in Fig. 2*C1* ,*C2*, this yields distinct but partly overlapping distributions, emphasizing the importance of both of these parameters for the classification of cells into either of the two cell types.

### Effect of serotonin on type I and type II cells

**Figure 3.**
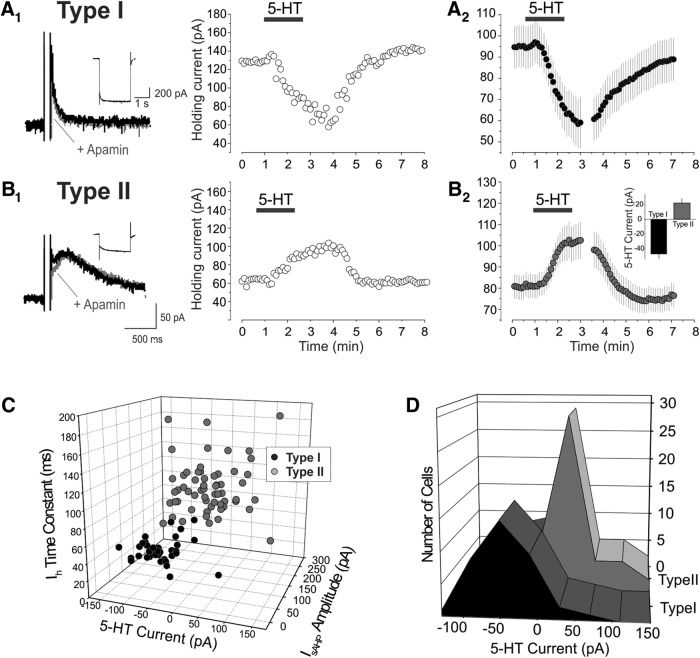
Effect of serotonin on type I and type II pyramidal cells of L5. ***A_1_***, Administration of serotonin (10 μm) to an electrophysiologically identified type I pyramidal cell of L5 elicits an inward current that recovers after removal of serotonin from the bath. *A*_2_, Summary plot illustrating the mean current (± SEM) elicited by serotonin on 38 type I cells of L5. Vh = –60 mV. ***B_1_***, Administration of serotonin (10 μm) to an electrophysiologically identified type II cell of L5 elicits an outward current that recovers on removal of serotonin from the bath. ***B_2_***, Summary plot illustrating the mean current (± SEM) elicited by serotonin on 59 type II cells tested. Vh = –60 mV. Notice that the initial outward current is followed a net inward current as previously reported for many of these cells (Araneda and Andrade, 1991). ***B_2_*** inset, Bar graph illustrating the peak initial current induced by serotonin. *, *p* < 0.001. ***C***, 3D plot graphing the time constant of I_h_, the amplitude of I_sAHP_, and the amplitude of the initial current induced by serotonin in the cell sampled in the current work. ***D***, Amplitudes distribution for the initial serotonin-induced current in type I and type II cells.

Previous work has shown that L5 pyramidal cells of the rat mPFC can also be divided based on their response to serotonin (Beique et al., 2007). Administration of serotonin to the medium-size pyramidal cells of L5 results in small, generally biphasic, changes in membrane potential or holding current (Araneda and Andrade, 1991; Beique et al., 2004, 2007). In contrast, administration of serotonin to the large pyramidal cells of deep L5 results in a strong depolarization/inward current that is often sufficient to initiate sustained spiking (Beique et al., 2007). To examine the relationship between serotonin responsiveness and the two cell types identified above, we administered serotonin (10 μm) to 38 type I and 59 type II cells in our sample. As illustrated in Fig. 3, type I cells generally responded to the administration of 5-HT with a strong inward current (Fig. 3*A*), whereas type II pyramidal cells generally responded to administration of serotonin with more modest changes in holding current generally involving an outward current that was frequently followed by a smaller inward current (Fig. 3*B*). Consistent with these observations, a histogram of the initial serotonin responses by cell type displays two distinct, though overlapping, distributions (Fig. 3*D*).

To better understand the relationship between electrophysiological properties and serotonin responsiveness, we plotted the amplitude of the initial response to serotonin, the amplitude of I_sAHP_, and the activation time constant for I_h_ for all the cells tested with serotonin. As illustrated in Fig. 3*C*, type I cells form a fairly tight cluster in terms of their electrophysiological properties and serotonin response. In contrast, type II cells form a much looser cluster, suggesting more heterogeneity in this cell population (Fig. 3*C*).

**Figure 4.**
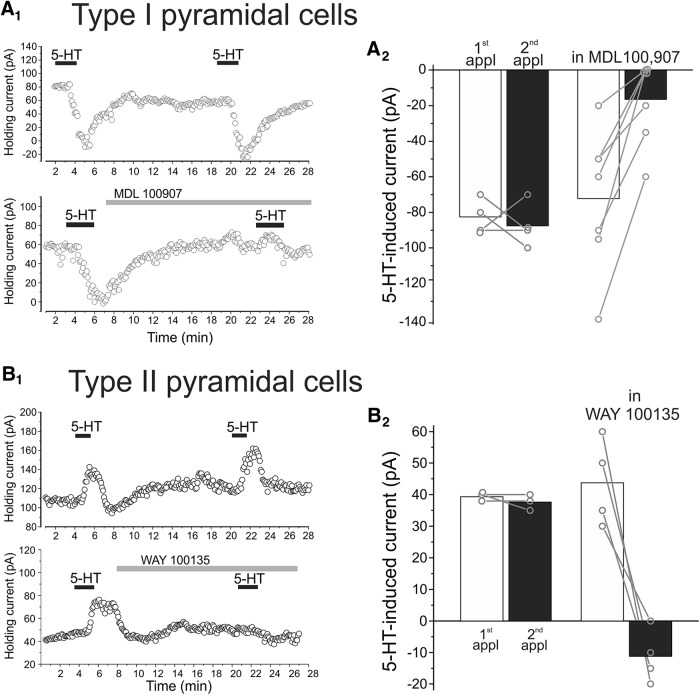
Pharmacology of serotonin responses in type I and type II L5 pyramidal cells of rat mPFC. ***A_1_***, Top, two consecutive applications of serotonin (10 μm) to a type I pyramidal cell results in comparable inward currents indicating minimal desensitization of the response. Bottom, administration of the selective 5-HT_2A_ receptor antagonist MDL100 907 (300 nm) between serotonin application inhibits the effect of a second serotonin application. ***A_2_***, Summary plot illustrating the effect of MDL100907 on the inward current elicit by serotonin on type I pyramidal cells. ***B_1_***, Top, two consecutive applications of serotonin (10 μm) to a type II pyramidal cell elicit comparable outward currents. Bottom, administration of the selective 5-HT_1A_ antagonist WAY100135 (3 μm) between serotonin application blocks the effect of a second application of serotonin. ***B_2_***, Summary plot illustrating the effect of WAY100135 on the outward current elicited by serotonin in type II pyramidal cells.

We next examined the pharmacology of the serotonin response in type I and type II cells. As illustrated in Fig. 4*A*_,_ two sequential administrations of serotonin to type I pyramidal neurons elicited comparable inward currents, indicating little or no desensitization of the response under our testing conditions. Administration of the 5-HT_2A_ selective antagonist, MDL 100907 (300 nM; Kehne et al., 1996), immediately after the first application greatly reduced or completely inhibited the serotonin-induced inward current (Fig. 4*A*, *p* < 0.01, *n* = 7 cells). These results indicate that the inward current induced by serotonin in type I pyramidal neurons of L5 is mediated predominantly if not exclusively by activation of receptors of the 5-HT_2A_ subtype. This is consistent with previous findings in the mPFC (Araneda and Andrade, 1991; Zhang, 2003; Beique et al., 2004, 2007; Avesar and Gulledge 2012).

Administration of serotonin to type II pyramidal cells generally resulted in outward currents that, in some cases, were followed by small inward currents. As illustrated in Fig. 4*B*, administration of the selective 5-HT_1A_ receptor antagonist WAY100135 (3 µM) completely blocked the ability of serotonin to elicit the outward current in these cells (first application, 43.75 ± 6.88 pA; second application, –11.25 ± 4.27 pA, *p* < 0.01, *n* = 4). In fact, in 3 of the 4 cells tested, a second administration of serotonin in the presence of WAY100135 resulted in a small inward current. These results indicate that the outward current induced by serotonin in type II pyramidal neurons of L5 is mediated by activation of 5-HT_1A_ receptor subtype, but that these cells also coexpress a second serotonin receptor. This second receptor has been reported to be of the 5-HT_2A_ subtype (Araneda and Andrade, 1991).

Previous work in rat mPFC has shown that 5-HT_2A_ receptor activation inhibits I_sAHP_ and induces the appearance of a I_sADP_ (Araneda and Andrade, 1991; Villalobos et al., 2005, 2011; Beique et al., 2007). Consistent with these earlier findings, administration of serotonin (10–30 µm) to L5 pyramidal cells displaying type II electrophysiological properties elicited an inhibition of I_sAHP_ (5 of 5 cells tested, not shown) and the appearance of an I_sADP_ (4 of 5 cells tested). Similarly, administration of serotonin to L5 pyramidal cells displaying type I electrophysiological properties elicited the appearance of an I_sADP_ (5 of 5 cells tested). We interpret these results to indicate that the two distinct subpopulations defined by their electrophysiological characteristics broadly correspond to the two distinct subpopulations defined by their serotonin receptor subtype expression.

### Type I and type II cells differ in their axonal projections and dendritic morphology

**Figure 5.**
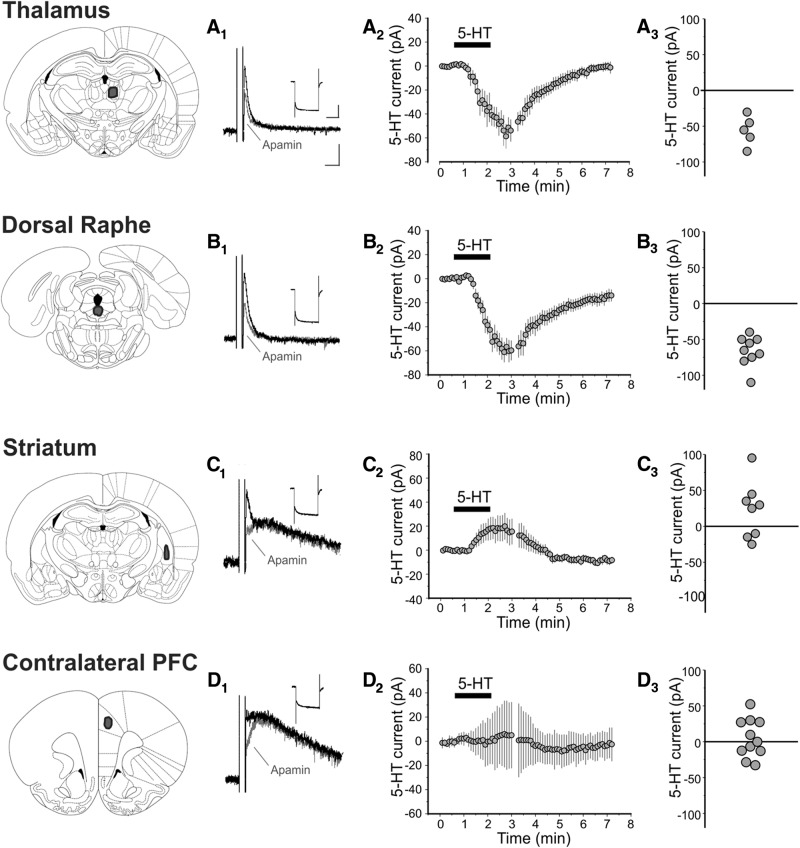
Effect of serotonin on retrogradely labeled L5 pyramidal neurons. Thalamus. ***A_1_***, I_sAHP_ and I_h_ recorded from a L5 neuron retrogradely labeled from the thalamus. I_h_ scale: 200 pA, 1 s. I_sAHP_ scale: 50 pA, 250 ms. The scale applies to panels ***A_1_–D_1_***. ***A_2_***, Summary plot illustrating the mean current (± SEM) elicited by serotonin (10 μm) in neurons retrogradely labeled from the thalamus (*n* = 5 cells). ***A_3_***, Graph illustrating the peak serotonin current recorded in these cells. Dorsal raphe. ***B_1_***, I_sAHP_ and I_h_ recorded from a L5 neuron retrogradely labeled from the dorsal raphe. ***B_2_***, Summary plot illustrating the mean current (± SEM) elicited by serotonin (10 μm) in neurons retrogradely labeled from the dorsal raphe (*n* = 9 cells). ***B_3_***, Graph illustrating the peak serotonin current recorded in these cells. Striatum. ***C_1_***, I_sAHP_ and I_h_ recorded from a L5 neuron retrogradely labeled from the striatum. ***C_2_***, Summary plot illustrating the mean current (± SEM) elicited by serotonin (10 μm) in neurons retrogradely labeled from the striatum (*n* = 8 cells). ***C_3_***, Graph illustrating the peak serotonin current recorded in these cells. Contralateral PFC. ***D_1_***, I_sAHP_ and I_h_ recorded from a L5 neuron retrogradely labeled from the contralateral PFC. ***D_2_***, Summary plot illustrating the mean current (± SEM) elicited by serotonin (10 μm) in neurons retrogradely labeled from the contralateral PFC (*n* = 11 cells). ***D_3_***, Graph illustrating the peak serotonin current recorded in these cells. The cells illustrated in panels ***A–D*** were assigned a probability of at least 95% for belonging to the type I class (***A*** and ***B***) or the type II class (***C*** and ***D***) by the Bayesian classifier.

As outlined above, pyramidal cells of L5 fall into two major classes, long-range corticofugal and intratelencephalic neurons. To determine if the two cell types identified above in rat slices correspond to these two cell classes, we injected a fluorescent retrograde tracer at four sites known to be projection targets for L5 pyramidal neurons of the PFC (Sesack et al., 1989; Gabbott et al., 2005). We then recorded from retrogradely labeled pyramidal cells in L5 of the prelimbic and anterior cingulate subdivisions of the mPFC. Cells projecting to the thalamus (*n* = 5, Fig. 5*A*) and dorsal raphe (*n* = 9, Fig. 5*B*) displayed the electrophysiological profile of type I pyramidal cells and responded to the administration of serotonin with strong inward currents (–56.00 ± 9.27 and –66.11 ± 7.01 pA, respectively). In contrast, pyramidal cells projecting to the striatum (*n* = 9, Fig. 5*C*) displayed the electrophysiological profile of type II pyramidal cells and responded to the administration of serotonin with an overall net outward current (Fig. 5*C*, 22.50 ± 13.82 pA). Pyramidal cells projecting to the contralateral PFC (Fig. 5*D*) also displayed the electrophysiological characteristics of type II pyramidal cells, but administration of serotonin produced little net current as a result of considerable cell-to-cell variability. We interpret these results to indicate that cells projecting subcortically correspond to type I pyramidal cells, whereas cells projecting to contralateral cortex and striatum (i.e. intratelencephalic pyramidal cells) correspond to type II pyramidal cells.

**Figure 6.**
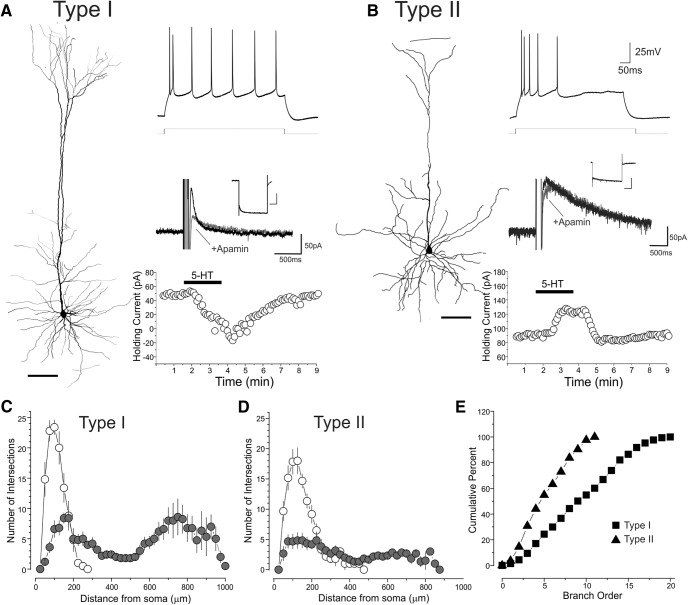
Neuronal morphology of type I and type II neurons. ***A***, Reconstruction of an electrophysiological and pharmacologically identified type I neuron. Neuron reconstruction scale: 100 μm, I_h_ inset scale, 500 ms, 200 pA. ***B***, Reconstruction of an electrophysiological and pharmacologically identified type II neuron. Neuron reconstruction scale: 100 μm, I_h_ inset scale, 500 ms, 200 pA. ***C***, ***D***, Scholl plots for the apical (filled circle) and basal (hollow circles) dendrites. Error bars depict the SEM. Branch order cumulative histogram for type I and type II pyramidal cells of L5. Panels ***C–E*** depict results derived from 5 type I and 7 type II neurons.

A number of previous studies have shown that long-range projecting corticofugal neurons and intratelencephalic projecting neurons also differ in terms of their dendritic morphology (e.g. Molnar and Cheung, 2006; Hattox and Nelson, 2007; Dembrow et al., 2010; Avesar and Gulledge, 2012; van Aerde et al., 2015). Therefore, we next examined whether electrophysiologically defined type I and type II cells in the mPFC also differed in terms of their dendritic arborizations. We recorded with biocytin-filled electrodes and tested and reconstructed 11 pyramidal cells, 5 of which were type I and 6 were type II neurons. As illustrated for two representative cells in Fig. 6*A*,*B*, type I and type II pyramidal neurons displayed distinctly different dendritic arbors. Most strikingly, type I cells exhibited a much larger and complex apical dendritic tree compared to the less extensively branched apical dendrites of type II cells. To quantitatively assess these differences, we conducted a 3D Sholl analysis on the reconstructed cells (Fig. 6*C*,*D*). This analysis indicated that type I cells exhibited a significantly greater number of intersections in the outer concentric spheres than type II cells (*F*_(1,90),_ *p* <0.0001). Because such crossings are made by the dendrites of the apical tuft, these results quantitatively confirmed the difference in the apical dendritic tree between these two cell types. The greater complexity of the apical dendrites of type I pyramidal neurons is also evident when plotting the cumulative number of branches as a function of branch order (Fig. 6*E*). Overall, these results indicate that type I and type II neurons display morphologically distinct apical dendritic trees, with type I pyramidal cells displaying more complex apical dendrites that terminate in a large, extensively branched tuft, whereas type II cells feature apical dendrites with fewer oblique branches and a sparse apical tuft. This provides additional support to the idea that type I cells correspond to long-range corticofugal neurons and type II cells correspond to intratelencephalic L5 pyramidal neurons.

### Effects of serotonin on type I and type II cells in mice

**Figure 7.**
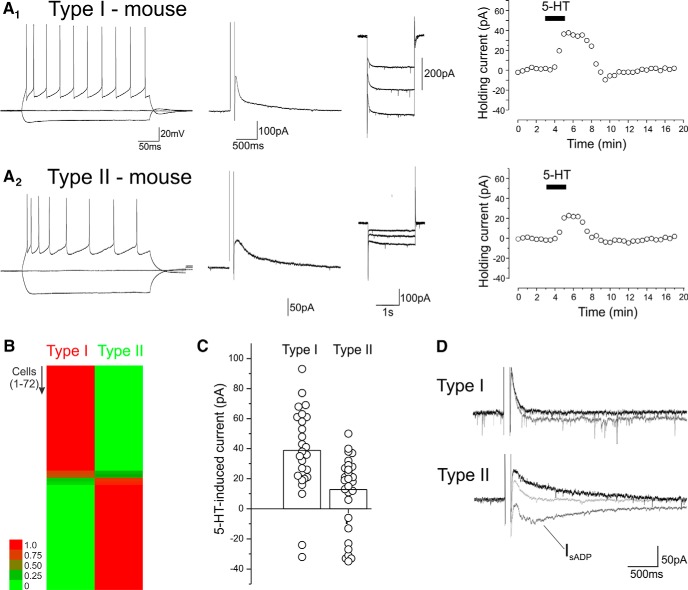
Type I and type II cells in L5 of the mouse mPFC. ***A_1_***, ***A_2_***, Electrophysiological characterization and response to serotonin administration (10 μm) in two L5 pyramidal cells, one classified as a type I and the other as type II by the Bayesian sorter. ***B***, Heat map depicting the probability that each of the 72 cells tested belongs to the type I and type II classes as determined by Autoclass. The probability for each cell is color-coded as depicted in the scale bar. ***C***, Summary graph plotting the amplitude of the peak serotonin-induced currents in the cells sampled. ***D***, Effect of serotonin on I_sAHP_ and I_sADP_ in type I and type II cells. Traces were baseline subtracted for clarity. The type I cell responded to serotonin with a 69-pA outward current and the type II cell with a –24-pA inward current. Overall, in this group of cells, serotonin (10–30 μm) elicited a –14 ± 6.5-pA inward shift in the aftercurrent in type I cells and –27 ± 3.5 pA in type II cells (*p* < 0.01).

These results outlined above, obtained in rats, differ significantly from those obtained in previous studies by Avesar and Gulledge (2012) and Stephens et al. (2014) using mice. In rats, we find that long-range corticofugal cells express 5-HT_2A_ receptors and are excited by serotonin, while intratelencephalic neurons express 5-HT_1A_ receptors, and secondarily 5-HT_2A_ receptors, which together remodel their excitability. In contrast, Avesar and Gulledge (2012) and Stephens et al. (2014) reported that corticopontine (i.e., long range corticofugal) neurons express predominantly 5-HT_1A_ receptors and are inhibited by serotonin, whereas commissural (i.e. intratelencephalic) cells express 5-HT_1A_ receptors and 5-HT_2A_ receptors and can be excited, inhibited, or express biphasic changes in firing in response to serotonin. To address this apparent discrepancy, we reexamined the effects of serotonin on pyramidal cells of the mPFC in brain slices derived from mice aged P35–P47 (*n* = 72 pyramidal cells in 13 mice). Consistent with findings by others (Hattox and Nelson, 2007), L5 pyramidal cells in mice cortex form a heterogeneous group in terms of spike frequency adaptation as well as I_sAHP_ and I_h_ when recorded in voltage clamp (Fig. 7*A*). As with rats, we used the unsupervised Bayesian clustering algorithm to sort L5 pyramidal neurons into classes. Because I_sAHP_ was smaller in mice, and appeared to be differentially expressed but still present to some extent in most cells, we used the ratio of I_mAHP_ and I_sAHP_ amplitudes and the kinetics of I_h_ as sorting variables. As illustrated in the heat map in Fig. 7B, this procedure sorted the recorded neurons into two classes analogous to the type I and type II classes present in rats, with most cells assigned to one class or the other with a high probability.

For the purpose of analyzing the effects of serotonin on these two cell classes, we excluded from the analysis cells that could not be assigned to either class with a probability of at least 90%. This resulted in the exclusion of 6 cells from our sample of 72 neurons. As illustrated in Fig. 7*A*,*C*, administration of serotonin (10 μm) elicited a net outward current in both type I, presumed long-range corticofugal, as well as type II presumed intratelencephalic neurons (38.9 ± 5.3 pA, *n* = 29, vs. 12.8 ± 3.7 pA). However it is worth noting that we observed considerable cell-to-cell variability, especially among type II cells. The serotonin-induced outward currents were blocked by WAY100135 or WAY100635, indicating that they were mediated by 5-HT_1A_ receptors (*n* = 11 cells).

As outlined above, in both rats and mice, activation of 5-HT_2A_ receptors inhibits I_sAHP_ and triggers the appearance of a slow I_sADP_ in pyramidal cells (Araneda and Andrade, 1991; Villalobos et al., 2005). In the current sample, administration of serotonin elicited the inhibition of I_sAHP_, often accompanied by its replacement by I_sADP_, in 18 of 28 type I cells and 28 of 29 type II cells tested (Fig 7*D*). This suggests that 5-HT_1A_ and 5-HT_2A_ receptors are most likely coexpressed by both cell types in mice, although clearly 5-HT_1A_ receptors predominate in long-range corticofugal cells. This interpretation is broadly consistent with previous reports (Avesar and Gulledge, 2012; Stephens et al., 2014).

### Effects of serotonin on mice L5 pyramidal cells during development

Previous studies in rats have shown that serotonin depolarizes and excites L5 pyramidal cells during the first 2 postnatal weeks (Zhang, 2003; Beique et al., 2004). Although these earlier studies did not distinguish between long-range corticofugal and intratelencephalic neurons, the invariant depolarization and excitation observed suggests this effect is common to both cell classes. Are the effects of serotonin on mice L5 pyramidal cells also developmentally regulated? To address this question, we recorded from 161 L5 pyramidal cells of the mPFC during the first postnatal month (P7–P26). During the early postnatal period, most if not all pyramidal neurons of L5 exhibit a large I_h_ with slow activation kinetics. This I_h_ does not appear qualitatively or quantitatively continuous with those seen at later developmental stages. This made it difficult to assign these cells to either of the cell types using the criteria used for older animals. Therefore we analyzed L5 cells as a single group.

**Figure 8.**
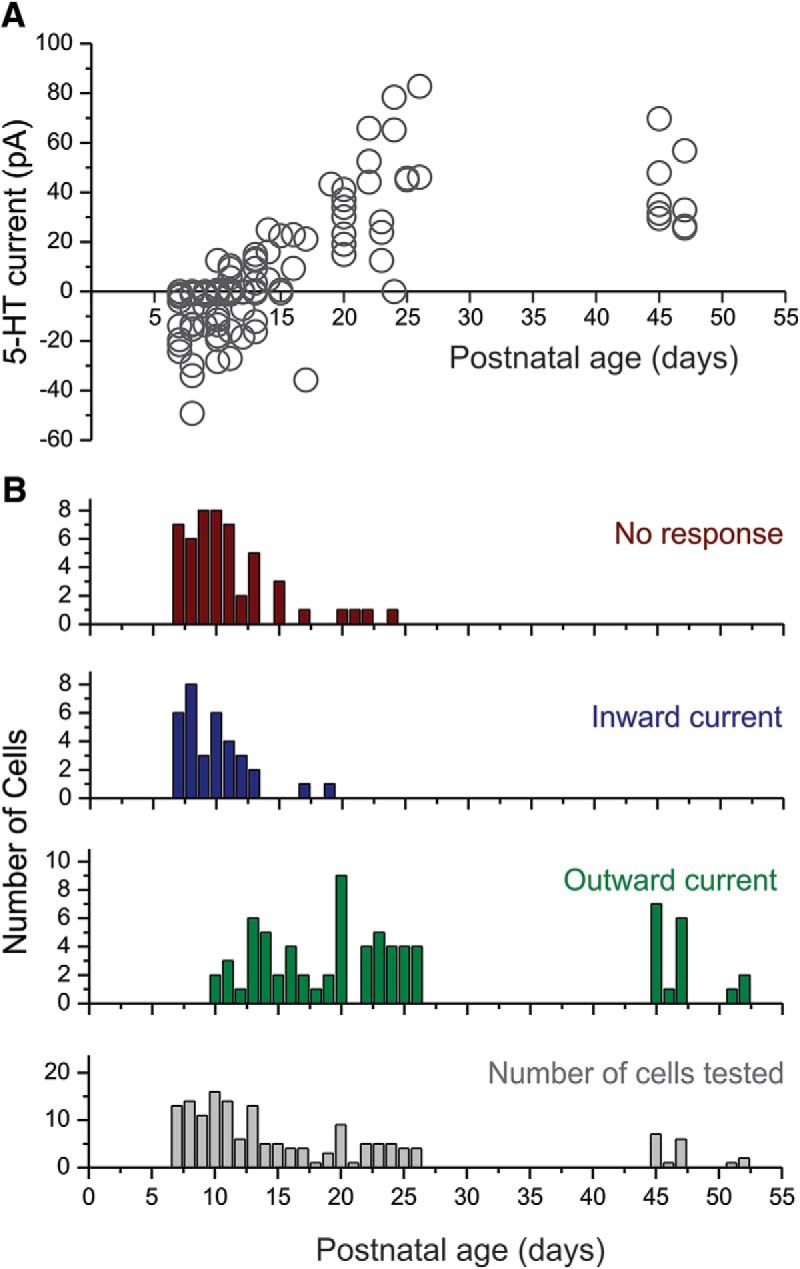
Effects of serotonin on mouse L5 pyramidal neurons during the early postnatal period. ***A***, Graph plotting the peak initial current elicited by serotonin on pyramidal cells of L5 as a function of postnatal age. ***B***, Histograms depicting the number of cells exhibiting no response, an inward current, and outward current as a function of postnatal age, as well as the number of cells tested at each age.

Fig. 8 illustrates the peak amplitude of the serotonin-induced current during the first postnatal month. As originally seen in rats, the response to serotonin depended on the age of the mouse. Bath administration of serotonin (30 μM, 1 min) triggered no effect or an inward current in slices derived from young pups (P7–P12). However, this effect progressively switched to an outward current at the end of the second week (Fig. 8), which then increased in amplitude to reach adult levels by P26. Interestingly, between P7 and P11, we could not detect any effect of serotonin in a large fraction of the L5 pyramidal neurons tested, an observation that contrasts with the more widespread effect of serotonin on L5 pyramidal neurons in rats (12 responders of 12 cells tested in the current study, inward current: –40.00 ± 8.68 pA, mean ± SEM). Thus there are similarities but also some significant differences in the trajectory of serotonin responses in rats and mice during development.

## Discussion

In the present work, we have investigated the effects of serotonin on the excitability of pyramidal cells of L5 in the rat and mouse mPFC. Rat L5 pyramidal cells of this region can be sorted into two main classes corresponding to long-range corticofugal and intratelencephalic neurons. We show that serotonin elicits a 5-HT_2A_ receptor–mediated inward (depolarizing) current in long-range corticofugal cells, while it elicits a more variable response dominated by a 5-HT_1A_ receptor–mediated (hyperpolarizing) outward current in intratelencephalic L5 neurons. We then show that these effects contrast with those seen in mice where serotonin responses in presumed long-range corticofugal as well as intratelencephalic L5 pyramidal cells are dominated by a 5-HT_1A_ receptor–mediated (hyperpolarizing/inhibitory) outward current. Finally we show that serotonin responses in L5 of the mouse mPFC are developmentally regulated with 5-HT_1A_ receptor–mediated outward currents not emerging until the end of the second postnatal week.

It has long been recognized that L5 is heterogeneous and contains two classes of neurons, intratelencephalic neurons that project within the telencephalon and long-range corticofugal cells that project to distal subcortical targets (Koester and O’Leary, 1993; Molnar and Cheung, 2006). More recent studies have shown that these two cell classes differ in terms of their developmental origin (Molyneaux et al., 2007; Leone et al., 2008) and their adult electrophysiological properties (Christophe et al., 2005; Hattox and Nelson, 2007; Dembrow et al., 2010; Groh et al., 2010; Avesar and Gulledge, 2012; Gee et al., 2012; van Aerde et al., 2015). In current clamp recordings, long-range corticofugal and intratelencephalic neurons can be seen to differ in terms of their spike frequency adaptation and the presence of a voltage sag in response to constant hyperpolarizing current injection. In the current work, we used an unsupervised Bayesian classifier to sort a sample of a hundred L5 pyramidal neurons based on the amplitude of I_sAHP_, a major contributor to spike frequency adaptation, and the time constant of I_h_, since the voltage sag would be most pronounced in cells expressing a fast I_h_. This classifier yielded two cell classes that we have called type I and type II pyramidal cells. Retrograde labeling and neuronal reconstructions confirmed that these two cell classes correspond to long-range corticofugal and intratelencephalic neurons, respectively. Overall, these results and classification are broadly consistent with previous work on L5 of the rat mPFC (Dembrow et al., 2010; van Aerde and Feldmeyer, 2015), although the robust sorting obtained using I_sAHP_ and I_h_ was unexpected.

Previous studies have shown that many pyramidal cells of L5 coexpress 5-HT_1A_ and 5-HT_2A_ receptors in the mPFC. Activation of the 5-HT_1A_ receptors in these cells elicits a hyperpolarization/outward current, while the activation of the coexpressed 5-HT_2A_ receptors elicits a delayed depolarization/inward current and the inhibition of the sAHP/I_sAHP_ (Araneda and Andrade, 1991). The coactivation of these two receptors by serotonin effectively remodels the integrative properties of these cells (Araneda and Andrade, 1991). Although these cells represent the most common cell type in L5, more recent work has identified a second population of cells in this area that appears to express solely 5-HT_2A_ receptors and is strongly excited by serotonin (Beique et al., 2007). In the current work, we show that the first of these cell types, which we call type II, corresponds to intratelencephalic neurons, and the second cell type, which we call type I, corresponds to long-range corticofugal pyramidal cells. These findings add to the growing appreciation of the differential effects of serotonin on pyramidal cell subtypes, an idea that can be traced to the prescient work by Spain in cat motor cortex >20 years ago (Spain, 1994). They also add to a growing literature documenting the differential regulation of long-range corticofugal and intratelencephalic L5 pyramidal cell types by neuromodulators (Shepherd, 2013).

The results outlined above, obtained in rats, differ dramatically from those obtained in mice by Gulledge and colleagues (Avesar and Gulledge, 2012; Stephens et al., 2014), who reported 5-HT_2A_ receptor–mediated excitations in commissural (i.e., intratelencephalic) neurons and 5-HT_1A_ receptor–mediated hyperpolarizations in both commissural and cortico-pontine (i.e., long-range corticofugal) pyramidal cells. To explore this discrepancy, we conducted recordings in L5 of the mouse mPFC under conditions identical to those we used in rats. We could again distinguish two electrophysiological cell types analogous to those present in rats that we interpret to correspond to intratelencephalic and long-range corticofugal neurons. Remarkably, we could confirm that in mice, 5-HT_1A_ and 5-HT_2A_ receptors are coexpressed in both cell types, with 5-HT_1A_ receptor–mediated responses predominating in long-range corticofugal cells and 5-HT_2A_ receptor–mediated depolarizations being vastly more robust in intratelencephalic neurons. This indicates that the effect of serotonin on long-range corticofugal cells is essentially reversed in rats and mice; a predominant 5-HT_2A_ receptor–mediated depolarization/inward current in rats and a predominant 5-HT_1A_ receptor–mediated hyperpolarization/outward current in mice.

In contrast, the work of our labs converges to demonstrate that the effect of serotonin on intratelencephalic neurons is similar in rat and mice and involves the coactivation of 5-HT_1A_ and 5-HT_2A_ receptors. Thus our work indicates that 5-HT_2A_ receptors are broadly expressed in rat and mouse L5 intratelencephalic neurons, where they increase membrane excitability by targeting multiple ion currents (Araneda and Andrade, 1991; Villalobos et al., 2005, and current work). Similarly, work by the Gulledge lab has shown broad expression of the predicted 5-HT_2A_ receptor–mediated activity-dependent excitations in mouse L5 intratelencephalic neurons of the mPFC (Avesar and Gulledge, 2012; Stephens et al., 2014). Similarly, both labs also concur in observing robust 5-HT_1A_ responses in these same cells, although our work suggests a somewhat higher prevalence of such responses. This modest quantitative discrepancy could reflect our reliance on measurements of individual membrane currents to assess receptor expression compared with the more integrative approach of examining firing rate. Alternatively, it could reflect differences in the animal strain used, how we define L5 in the slice, or even differences in our recording conditions.

Previous studies in rat have shown a strong developmental regulation of the effects of serotonin in the immediate postnatal period. Specifically serotonin broadly excites rat L5 pyramidal cells after birth, and it is not until the third postnatal week, after opening of the eyes, that the intratelencephalic neurons begin to express serotonin-induced 5-HT_1A_ receptor–mediated inhibitory responses (Zhang, 2003; Beique et al., 2004). Here we show a similar developmental regulation of serotonin responses in pyramidal cells of L5 of the mouse mPFC. As in rats, serotonin elicits predominantly excitatory responses during the early postnatal period, and 5-HT_1A_ receptor–mediated hyperpolarizing responses do not begin to appear until the second postnatal week. The main difference between rats and mice is the presence of a large proportion of L5 pyramidal cells that appears unresponsive to serotonin during early postnatal development. During these experiments, we considered the possibility that these nonresponsive cells may represent long-range corticofugal neurons. Unfortunately, it was difficult to identify immature intratelencephalic from long-range corticofugal cells based on their electrophysiological properties. Therefore we could not readily test for differences in the developmental trajectory of these two cell classes.

The results outlined above clarify how serotonin regulates the output of the mPFC. In both rats and mice, serotonin regulates intratelencephalic L5 output by activating 5-HT_1A_ and 5-HT_2A_ receptors which, when coactivated on single neurons, modulate the input-output relationship of these cells (Araneda and Andrade, 1991). Serotonin also regulates the long-range subcortical output of L5 neurons but appears to do so differently in rats and mice. In rats, serotonin acts via 5-HT_2A_ receptors to facilitate the output of cortex via L5 long-range projecting corticofugal cells, whereas in mice it acts via 5-HT_1A_ receptors to inhibit this output. This opposite effect of serotonin on L5 output in rat and mouse was unanticipated. It is possible, perhaps even probable, that this difference may be rooted in as yet unrecognized ethological differences between these two rodent species. In other words, this may represent a real species difference. Alternatively, because the rats in our experiments were purchased from a vendor while the mice were bred in-house, it is also possible that this difference could reflect their divergent developmental histories. Early stress, for example, has been shown to greatly facilitate 5-HT_2A_ receptor function in the rat mPFC (Benekareddy et al., 2010). Additional studies will be needed to distinguish between these possibilities.

## Synthesis

Reviewing Editor: Douglas Bayliss, University of Virginia School of Medicine Decisions are customarily a result of the Reviewing Editor and the peer reviewers coming together and discussing their recommendations until a consensus is reached. When revisions are invited, a fact-based synthesis statement explaining their decision and outlining what is needed to prepare a revision will be listed below. The following reviewer(s) agreed to reveal their identity: Robert Foehring, Allan Gulledge.

This manuscript was seen by two reviewers who were positive about the contribution the paper makes to understanding 5-HT effects on different types of cortical pyramidal neurons, and with resolving species differences in those effects. They each suggest a number of minor points in presentation and discussion of the results that should be addressed. These points are presented in their comments appended below.

Reviewer #1

Advances the Field:

This is a solid paper that resolves discrepant findings for the pattern of 5HT response between different types of PN in L5 of prefrontal cortex. While many of the findings are confirmatory, they clearly show a species difference in the responses, ad an unbiased method that confirms the cell types based upon morphology, physiology, and projections, and clearly show how 5HT responses change with age in mice.

Comments to the Authors:

This study seeks to resolve discrepancies previously reported between labs for the effects of 5HT on L5 pyramidal neurons (PNs) in rat vs. mouse. The authors first combined use of whole cell current- and voltage-clamp recordings of L5 pyramidal neurons (PNs) from slices of prefrontal cortex of rats with retrograde labeling based upon projection target and anatomical reconstruction to confirm the presence of two populations of PNs in that layer. Type I PNs were large cells that projected subcortically and had more complex apical dendrites and apical tuft. Type II PNs were smaller, had simpler apical dendrites, and projected to contralateral cortex and striatum. They also confirmed characteristic electrophysiological properties for these two PN types - they differed in input resistance, IH kinetics, and the size of IsAHP. Type I PNs had very little IsAHP and this corresponded with little spike frequency adaptation (SFA) in these PNs. Type II PNs had large IsAHP and much greater SFA. Importantly, the results of an unbiased Bayesian classifier separated the PNs into two groups that corresponded closely to Type I and Type II defined by projections and morphology.

The effects of 5HT were then examined for these two types of PNs in rats. The results confirmed earlier findings in rats by the Andrade lab in that 5HT elicited inward currents in Type I PNs vs. an initial outward current in Type II PNs. In Type II PNs, the initial outward current was followed by a small inward current. Also confirmed was the pharmacology of these responses (5HT1A for the outward current and 5HT2A for the inward currents). This pattern of 5HT effects was the same whether the two PN types were defined by projection, electrophysiology, or Bayesian statistics. The authors then repeated the experiments in mice and found a different pattern of response to 5HT: Type I PNs primarily showed outward currents and Type II PNs had outward followed by inward currents. These results basically confirmed previous findings in mice by the Gulledge lab. Finally, mice of different ages were tested to see if a shift in response type occurs with age, as previously shown in rats (initial inward currents in all cells shifting to the adult pattern (inward in Type I and initial outward in Type II). At the earliest ages, 5HT elicited inward current in all mouse PNs. With increasing age, a shift occurred to primarily outward currents. Thus mice and rats differ in the pattern of the dominant 5HT response vs. PN type as well as the details of the developmental shift in 5HT response type.

This is a solid paper. It is very clearly written and the data and figures are of high quality. The results are convincing and go a long way towards resolving the controversy in the literature about 5HT responses vs. PN type. The results suggest that the difference is either a real species difference or is otherwise due to the specific genetic history of the mice and rats used.

I have a few criticisms of the organization of Figures 1 and 2, the choice of “slow adapting” vs. “fast adapting” to describe the two electrophysiological PN types, and some of the functional conclusions in the Discussion. The text considers L5 PNs as a whole for an initial description of electrophysiological differences between PNs (IsAHP, input resistance, adaptation index, and IH time constant) and then proceeds to a quantitative description of the properties of two PN types. Figure 1A,B are a qualitative description of two adaptation types and Figure 1C1 illustrates the distribution of adaptation index for the whole population of PNs. In contrast, the inset in Figure 1C1 and Figures 1C2 and 1C3 separately present data for PNs of two defined types (i.e., as in Figure 2). This leads to some confusion in reading the text that could be alleviated by moving the inset of Figure 1C1 and 1C2 and 1C3 to Figure 2 (which would be in sequence with the order these data are discussed in the text).

From Figure 1A and 1B, it is not clear that the distinction in adaptation is between fast vs. slow and no kinetic data are provided to back up the “fast” vs.“slow” classification. Rather, it looks like Type I PNs show very little SFA (i.e., they are weakly adapting) and Type II PNs show strong SFA. Also, in the text an Adaptation Index is described but in Figure 1C1 the X axis is labeled “Accomodation Index”. The axis label should be changed to be consistent with the text.

Discussion of the overall functional effects of 5HT1A vs. 5HT2A receptors on Type I vs. Type II cells only considers the initial changes in inward vs. outward current (see Lines 397-399, 490-493, 519-522) and neglects the effects of 5HT on the sAHP and sADP. The sAHP is strongly modulated by 5HT2A receptors in Type II but not Type I PNs. Thus one would expect that whether 5HT increases or decreases excitability would depend on the type and amplitude of the stimuli.

Minor

1. Line 159: What is meant by “... most neurons were imaged in two identical overlapping stacks.”? If the stacks are truly identical they would completely overlap and it is unclear why this would be important to do. By “identical” are the authors referring to the parameters used to create stacks of two overlapping regions of the cell / slice?

2. Line 172: Was striatal injection of lumafluor beads ipsilateral or contralateral to the recorded cell in cortex (The Reiner lab has described differences between IT- and PT-type PNs in projections to ipsi- vs. contralateral striatum)?

3. Figure 1C1: X axis should be “Adaptation Index”, not “Accomodation Index”.

4. Lines 213-214 and Figure 1A,B: It is not clear in the figure that the SK-mediated ImAHP is the same in both cell types. Was this quantified?

5. Lines 305-306: How many cells showed these effects (vs. how many tested)? It would be appropriate to include these data as done later for mouse. Also, did 5HT induce a sADP in Type I as well as Type II?

6. Lines 374-376: The authors should clarify what is mean by IsAHP being less well defined in mouse. In the figure it looks like both Type I and II had IsAHP and in the example it is smaller in Type II than shown for rat.

7. Line 389: “n >8” is vague - how many cells were tested?

8. Lines 393-395: Since Figure 7 shows an IsAHP in mouse Type I cells, was it modulated by 5HT?

9. Lines 408-410: What about the electrophysiological properties of young animals precludes identification of cell classes?

Reviewer #2

Advances the Field

The paper reveals a species-dependency on 5-HT signaling.

Comments to the Authors

The report, “Serotonin differentially regulates L5 pyramidal cell classes of the medial prefrontal cortex in rats and mice”, describes differences in the cell-type-specificity of serotonergic signaling in projection neurons in the medial prefrontal cortex of two rodent models (rats and mice). The results are interesting in showing a species dependence of 5-HT actions of specific classes of cortical output neurons (corticofugal and intratelencephalic). The strong points of this paper are the comparison across two species, and the development of a novel method to classify projection cell classes based on physiological parameters. A challenge in interpretation is that this study uses different methodologies to measure 5-HT responses relative to some earlier works. However, using consistent methodology across species, the authors reveal clear and notable differences in the cell-type-specificity of 5-HT signaling in the prefrontal cortex of rats and mice that will be of interest to the field. This reviewer has mostly minor comments on the paper.

Main comment:

A key goal of this paper is to harmonize different findings in the literature regarding 5-HT signaling in the cortex. While going a long way toward that goal, a challenge is that different methodologies have been used by different laboratories. The authors should point out and discuss these methodological differences. For instance, in the current study, the authors use voltage-clamp and relatively prolonged (minutes) exposures to 5-HT, whereas some other studies (e.g., Avesar and Gulledge, 2012) have measured responses to transient 5-HT application in current clamp. Given that the 2A response is voltage-dependent (e.g., Araneda & Andrade, 1991), the use of voltage-clamp measures might influence the magnitude and/or likelihood of observing a 2A response in a given neuron. Could some of the variability of responses in type II neurons reflect voltage clamping at a relatively hyperpolarized membrane potential? Some of these small responses would no doubt be larger if neurons were allowed to depolarize. Similarly, the voltage clamp will be less effective in larger, type I neurons, raising the question whether poor space clamp may explain the larger peak current amplitudes in these neurons? As a rough way to test for this, the authors could see if there a relationship between size of 5-HT responses and input resistance within each class of neuron. Other variables to consider might be temperature of recording and intracellular solutions used during recordings.

Minor comments:

1. The last line of the abstract (and significance statement) may be misleading. Rather than “clarifying” the effect of 5-HT on pyramidal neurons, the results of this paper actually do a service by demonstrating the diversity of 5-HT signaling in various classes of projection neurons across species. This actually stimulates additional questions about 5-HT that the authors allude to in their final lines of the discussion.

2. It would be nice to show in Figure 5 where the unsupervised Bayesian classifier puts these neurons (% confidence) and where they would fall in Fig. 2A. Perhaps they could be added to Fig. 2A and color coded according to projection location.

3. The inclusion of the Rn comparison in Fig. 1C is somewhat confusing, as it is not clear without reading the legend that C1 is unsorted data. As the color is the same as the “type II” data in the other parts of panel C, one may wonder where the type I data are in C1.

4. It would be worth showing in a figure the 5-HT effect on sAHP and sADP in rats and mice.

5. Were animals of both sexes? What strain of mouse was used? These details should be in the methods.

6. Were the striatal injections ipsilateral? If so, it would be worth discussing which type of neuron (type 1 or 2) would be most likely labeled by such an injection based on past literature.

7. How similar were Ih taus across the range of -70 to -110 mV? If they vary across voltage, at which voltage steps were taus calculated for comparisons?

8. In Figure 8, what type of neurons are those out at 45-50 days old?

9. The authors state the input resistance was measured based on the “maximum” voltage deflection. By definition, this really should be steady-state voltage deflection, and for practical reasons they may find better sorting using such methodology, as the Ih-sag in type 1 neurons will tend to enhance the difference in input resistance between type I and type II neurons.

10. Excessive capitalization in lines 316 and 317.

11. The statement on line 333 should have some references added, and likely should describe how the author's data are consistent with those previous works.

1. The last line of the abstract (and significance statement) may be misleading. Rather than “clarifying” the effect of 5-HT on pyramidal neurons, the results of this paper actually do a service by demonstrating the diversity of 5-HT signaling in various classes of projection neurons across species. This actually stimulates additional questions about 5-HT that the authors allude to in their final lines of the discussion.

2. The authors should point out and discuss methodological differences between their present study and studies by others (notably Avesar & Gulledge, 2012, and Stephens et al., 2014). For instance, in the current study, the authors use voltage-clamp and relatively prolonged (minutes) exposures to 5-HT, whereas some other studies have measured responses to transient 5-HT in current clamp. Given that the 2A response is voltage-dependent (e.g., Araneda & Andrade, 1991), the use of voltage-clamp measures might influence the magnitude and/or likelihood of observing a 2A response in a given neuron. Could some of the variability of responses in type II neurons reflect voltage clamping at a relatively hyperpolarized membrane potential? Some of these small responses would no doubt be larger if neurons were allowed to depolarize. Similarly, the voltage clamp will be less effective in larger, type I neurons, raising the question whether poor space clamp may explain the larger peak current amplitudes in these neurons? As a rough way to test for this, the authors could see if there a relationship between size of 5-HT responses and input resistance within each class of neuron.

3. It would be nice to show in Figure 5 where the unsupervised Bayesian classifier puts these neurons (% confidence) and where they would fall in Fig. 2A. Perhaps they could be added to Fig. 2A and color coded according to projection location.

4. The inclusion of the Rn comparison in Fig. 1C is somewhat confusing, as it is not clear without reading the legend that C1 is unsorted data. As the color is the same as the “type II” data in the other parts of panel C, one may wonder where the type I data are in C1.

5. It would be worth showing in a figure the 5-HT effect on sAHP and sADP in rats and mice.

6. Were animals of both sexes? What strain of mouse was used? These details should be in the methods.

7. Were the striatal injections ipsilateral? If so, it would be worth discussing which type of neuron (type 1 or 2) would be most likely labeled by such an injection based on past literature.

8. How similar were Ih taus across the range of -70 to -110 mV? If they vary across voltage, at which voltage steps were taus calculated for comparisons?

9. In Figure 8, what type of neurons are those out at 45-50 days old?

10. The authors state the input resistance was measured based on the “maximum” voltage deflection. By definition, this really should be steady-state voltage deflection, and for practical reasons they may find better sorting using such methodology, as the Ih-sag in type 1 neurons will tend to enhance the difference in input resistance between type I and type II neurons.

11. Excessive capitalization in lines 316 and 317.
